# CRISPR and transposon in vivo screens for cancer drivers and therapeutic targets

**DOI:** 10.1186/s13059-020-02118-9

**Published:** 2020-08-19

**Authors:** Imran Noorani, Allan Bradley, Jorge de la Rosa

**Affiliations:** 1 Department of Medicine, University of Cambridge School of Clinical Medicine, University of Cambridge, Cambridge Biomedical Campus, Cambridge, CB2 0QQ UK; 2 Department of Neurosurgery, University of Cambridge, Cambridge, CB2 0QQ UK; 3 The Wellcome Trust Sanger Institute, Wellcome Trust Genome Campus, Hinxton, Cambridgeshire, CB10 1SA UK

## Abstract

Human cancers harbor substantial genetic, epigenetic, and transcriptional changes, only some of which drive oncogenesis at certain times during cancer evolution. Identifying the cancer-driver alterations amongst the vast swathes of “passenger” changes still remains a major challenge. Transposon and CRISPR screens in vivo provide complementary methods for achieving this, and each platform has its own advantages. Here, we review recent major technological breakthroughs made with these two approaches and highlight future directions. We discuss how each genetic screening platform can provide unique insight into cancer evolution, including intra-tumoral heterogeneity, metastasis, and immune evasion, presenting transformative opportunities for targeted therapeutic intervention.

## Introduction

Large-scale human tumor sequencing projects have been invaluable in establishing the genetic landscapes of many cancers [1]. These have identified hundreds of recurrently mutated genes, as well as copy-number variations, transcriptional changes, and epigenetic alterations, but the role of most of these alterations in oncogenesis is unknown. Cancer genomics primarily inform which mutations are present in cancer but have limited power to tell us which of these are functionally important [2, 3]. Therefore, complementary approaches are required to understand the functional role of such genetic changes, particularly of those that occur less frequently (so-called long-tail mutated genes) and of non-mutated putative cancer genes, and importantly how genetic drivers cooperate with one another for cancer evolution.

Early insertional mutagenesis screens in mice used retroviruses, yet viral tropism precluded their widespread use for cancer investigation [4, 5]. A fruitful approach over the last decade to pinpoint genetic drivers of cancer is through in vivo transposon mutagenesis, forward genetic screening in mice. DNA transposons are genetic elements that can shift positions within a genome. *Sleeping Beauty* (SB) and, more recently, *piggyBac* (PB) mutagenesis have been deployed to elucidate the functional drivers of many cancers, unveiling molecular changes underpinning cancer initiation, progression, metastasis, and resistance to treatment [6, 7].

Clustered regularly interspaced short palindromic repeats (CRISPR)/CRISPR-associated protein 9 (Cas9) is a prokaryotic, adaptive immune system that was adapted as a genome-editing tool in eukaryotic cells [8, 9]. CRISPR screens provide a complementary approach to transposon mutagenesis and RNAi screens for uncovering genetic mechanisms of cancer evolution. In recent years, CRISPR techniques have evolved to enable screening strategies to be performed in vivo [10, 11]. The simplicity by which gene editing can be performed and the low off-target effects have made CRISPR particularly attractive as a platform for genomic screens. Alternative variants of the Cas nuclease have afforded the ability to conduct gain- and loss-of-function screens for defining oncogenes, tumor suppressor genes (TSGs), therapeutic vulnerabilities, and immunotherapy targets [12–15], as well as to functionalize the non-coding genome.

In this review, we focus our discussion on recent developments in transposon mutagenesis and CRISPR cancer screens in vivo, and the relative advantages of these complementary tools for discovery of cancer genes. We highlight future directions for the field to maximize the translational impact of these powerful techniques in order to develop a comprehensive functional understanding of cancer genes, including their role in tumor evolution and metastasis, and to reveal opportunities for therapeutic intervention.

## Transposon mutagenesis screens in cancer

DNA-transposons are genetic elements that move through the genome by a “cut-and-paste” mechanism and are generally inactive in mammalian cells in nature [16]. However, through genetic engineering, several groups have generated active recombinant transposons that can be used as insertional mutagens in mice and other vertebrates [17–19]. They consist of two components: the transposon vector and the transposase enzyme. When these are present in the same cell, the transposase recognizes the terminal repeats of the transposon and excises it from the donor locus. The transposon can then insert itself elsewhere in the genome creating a mutation and be used as a tag to pinpoint the genes it mutates.

**Fig. 1:**
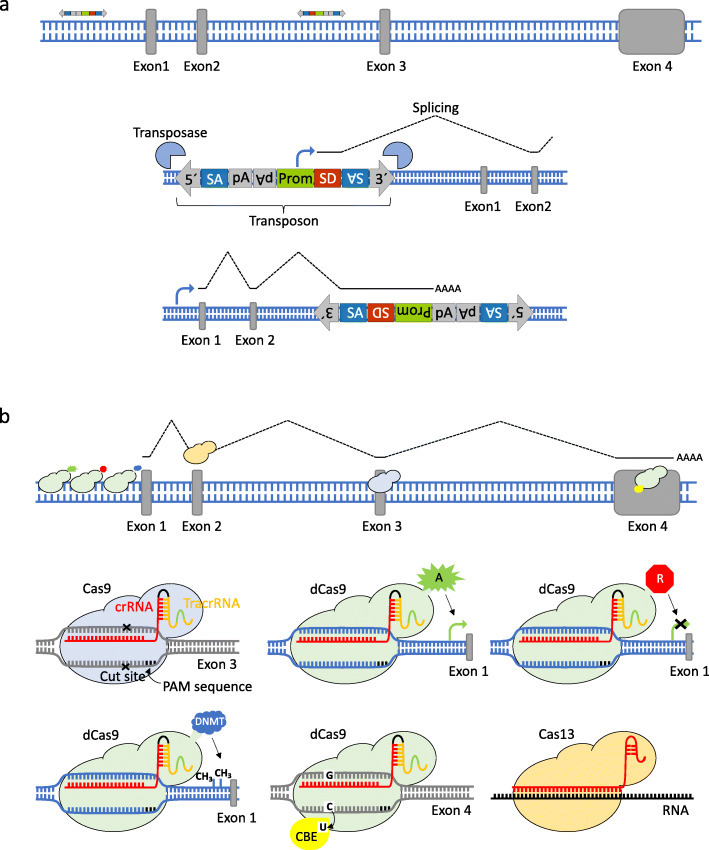
Transposon mutagenesis and CRISPR-induced genetic and epigenetic alterations. **a** *Sleeping beauty* (SB) and *piggyBac* (PB) transposons carry a promoter upstream of a splice donor (SD) sequence that can lead to overexpression of downstream genes. This is equivalent to activation of proto-oncogenes. Transposons also contain splice acceptors (SAs) followed by bidirectional polyadenylation (polyA) signals to cause termination of transcripts that arise when inserted into the body of a gene (primarily in introns). This is important for inactivating tumor suppressor genes (TSGs). **b** CRISPR-Cas9 is a two component system: the single guide RNA (sgRNA) directs the Cas9 nuclease to a complementary sequence in the genome where Cas9 will induce a double-strand break (DSB). The target genomic locus must be followed by a 5′-NGG-3′ motif (protospacer adjacent motif, PAM) for Cas9 to function. DSBs are resolved by non-homologous end-joining, or by homology directed repair (HDR) in the presence of a DNA repair template, which can be exploited to introduce precise genetic modifications or exogenous sequences. Other variants of the Cas nuclease have been engineered to function in mammalian cells, including a nuclease-deficient Cas9 (dCas9) fused to various effector domains: a transcriptional activator (A) for activation of gene expression, a transcriptional repressor (R) to knockdown gene expression [20], epigenetic effectors such as DNA methyltransferases (DNMT) for methylation, and base editors such as cytosine base editors (CBE) for generating point mutations

For cancer screens, transposons have been equipped with genetic elements that enable them to induce gain-of-function or loss-of-function mutations, depending on their orientation and position relative to the target gene (Fig. 1a). To perform insertional mutagenesis screens in mice, two types of mouse lines were engineered: “transposon mice” containing various transposon copies (ranging from one to a few hundred) on a single chromosome; and “transposase mice” that express the transposase in a constitutive or tissue-specific manner. By predisposing to cancer initiation using a mouse transgenic line carrying a known tumorigenic allele, such as a *Trp53* mutation [21], and then crossing in transposon and transposase alleles, one can map the subsequent transposon “common insertion sites” (CIS; genes “hit” more frequently than would be expected by chance) of resulting tumors to elucidate the functional mutations that cooperate with the predisposing mutation to drive cancer. With SB and PB, this was initially done using a constitutive approach (with SB or PB expressed throughout the body), giving rise to multiple cancers in the same mice [22, 23]. Conditional SB, and more recently PB, conditional screening strategies have successfully enabled exploration of tissue-specific cooperating groups of genes in several cancers [24–32]; these screens typically use Cre-transgenic mouse lines to express the transposase in target tissues.

SB and PB are the most widely used transposons for cancer screens. SB transposons derive from the salmonid *Tc1-Mariner* elements [17]. Modifications to the terminal repeats and transposase were made early on in order to improve transposition efficiency [18, 33, 34]. SB integrates into TA dinucleotides that are duplicated upon integration and leave behind a 2–5-bp “footprint” mutation after mobilization [35]. Thus, insertions that were driving oncogenesis can still be identified through exome or genome sequencing even if the transposon has subsequently mobilized into another site [24]. However, footprints lead to varying degrees of genetic disruption, contributing to “noise” in the screen. SB also has a tendency for “local hopping,” that is, excision and reintegration of the transposon into a neighboring region of the same chromosome. This is a disadvantage for cancer screens because it leads to passenger mutations near cancer genes [36], and must be considered when analyzing screening data [37, 38]. Further characteristics of the SB system have been reviewed in detail elsewhere [19, 39–41].

The PB transposon is naturally found in the cabbage-looper moth *Trichoplusia ni* [42]. Efforts were made to adapt PB for mammalian systems [42], and specifically for forward genetic screens of cancer both in vitro and in vivo [36, 43, 44]. The higher transposition efficiency, larger cargo capacity, and lower rate of local hopping in comparison with SB make PB particularly useful for genome-wide screening. PB has a preference for inserting into TTAA sites [45] and does not leave a footprint after mobilization unlike SB. The increased tendency for PB to insert into open chromatin regions [43, 46, 47] relative to SB enables the identification of non-coding regions that may contribute to cancer. For example, a novel *Cdkn2a*-cis regulatory region was identified as a contributor to pancreatic cancer development by PB insertions [48].

Altogether, PB and SB are complementary tools with the deployment of both being more likely to lead to saturating mutagenesis and maximal gene discovery in cancer [48].

## CRISPR screens in cancer

Technologies for genetic manipulation of mammalian genomes based on engineered nucleases have evolved rapidly over the last decade. The CRISPR-Cas9 system consists of two components, the endonuclease Cas9 from *Streptococcus pyogenes* (SpCas9) and a single guide RNA (sgRNA). Cas9 is directed to cleave any desired genomic locus complementary to a unique sgRNA sequence. Thus, one can change the genomic target of Cas9 by simply changing the sgRNA sequence [49] (Fig. 1b). Introduced double-strand breaks (DSBs) can be repaired by error-prone non-homologous end joining (NHEJ), leaving small insertions and deletions (indels) which often cause loss of function of the target gene [50]. The co-expression of Cas9 and multiple sgRNAs within the same cell (multiplexing) enables not only the induction of more complex genomic alterations [51] (i.e., translocations, inversions, duplications, and large deletions), but also delineation of complex combinatorial gene networks [10, 52, 53].

Besides Cas9, other RNA-guided DNA endonucleases have been engineered with different features, as well as RNA-guided ribonucleases to manipulate cellular transcripts [54]. Moreover, a nuclease-deficient Cas9 (dCas9) has been fused to a variety of effector domains: transcriptional activators (e.g., VP64) to mediate gene activation (CRISPRa) [55], transcriptional repressors (e.g., KRAB) to knockdown gene expression (CRISPRi) [20], DNA methyltransferases (e.g., DNMT3A or TET1) or histone-modifying enzymes (e.g., p300) for regulating the epigenome [56], and base-editors (e.g., AID) for precise single-nucleotide exchanges [57–59]. Such base editors enable the introduction of point mutations in DNA (or RNA) without making DSBs, therefore not requiring NHEJ and leaving no indels [60]. Although DNA editing can also be achieved by supplying a donor DNA template whilst inducing a DSB with Cas9, this relies on homology-directed repair (HDR) which is mainly limited to G2 and S phases of the cell cycle and has low efficiency; efforts to increase the efficiency of HDR for DNA editing may facilitate its use for in vivo screens.

A number of studies have demonstrated the usefulness of CRISPR for genome-wide and focused cancer screens in vitro [55, 61–64]. In such studies, cancer cells are transduced with customized lentiviral libraries carrying up to thousands of sgRNAs against any genes of interest. After biological selection for a phenotype, such as resistance to a drug, DNA from cells is sequenced to define enriched (positive selection screen) and/or depleted (negative selection screen) sgRNAs driving or preventing this phenotype respectively. Such screens have enabled exploration of genetic underpinnings of many cancer phenotypes, including genes essential for cancer cell survival and growth [61, 65, 66], modifiers of drug resistance [64, 67], and synthetic lethal genetic interactions [68, 69]. RNAi screens have also been used for investigating cancer phenotypes in vivo, in particular by using lentiviral or retroviral transduction of short hairpin RNAs (shRNAs) to achieve genetic knockdown [70]. Although pooled RNAi screens have been instrumental in functional interrogation of cancer genes and drug resistance mechanisms [71], CRISPR provides generally fewer off-target effects compared with RNAi and also extra versatility for genetic manipulation, including not only knockdown, but knockout, activation, methylation, and base editing [72]. Detailed comparisons of CRISPR and RNAi screens have been reviewed elsewhere [73].

Despite the success of CRISPR in vitro screens, an in vivo approach is needed to account for interactions between cancer and host cells, including the immune microenvironment [74, 75]. The revolutionization of cancer treatment by immunotherapies underscores the importance of fully recapitulating tumor microenvironments which can only be accomplished in vivo. In the same way that a CRISPR-induced mutagenized pool of cells can be propagated in cell culture, it can also be implanted into mice for in vivo screens. These transplant models are attractive because of the comparative ease of gene editing with CRISPR in vitro prior to establishing an in vivo context. However, these models do not give rise to de novo tumors from normal cells at the organ site in situ, and they usually rely on immunodeficient host mice to facilitate the engrafting efficiency.

Direct in vivo mutagenesis of somatic cells provides a more faithful recapitulation of human cancer development than transplant models [76]. In these autochthonous mouse models, tumor cells originate de novo from endogenous cells in the target tissue and evolve towards malignancy in their natural microenvironment, under the surveillance of the host immune system. Moreover, induced mutations accumulate in a defined and uniform host genetic background, which aids the interpretation of their functional role in cancer.

Transposon mouse models provide an autochthonous setting for cancer development, but for CRISPR screens to be performed in these conditions, they require Cas9 and sgRNAs to be delivered directly to the target tissue. To facilitate this, transgenic mice expressing Cas9 in the germline, constitutively or in a tissue-specific (Cre-dependent) manner, have been developed [77]. These models can also overcome immunogenicity of Cas9 when this is introduced into mammals [78]. Initial studies with CRISPR-Cas9 in vivo focused on targeting one to three key cancer genes and provided proof of principle for the use of CRISPR to directly induce cancer in vivo. To date, efficient delivery of sgRNA libraries to different tissues of adult mice has been achieved through several methods, allowing for high-throughput discovery and validation of cancer genes in vivo.

A simple way to deliver CRISPR libraries into cells in vivo is through DNA transfection. For example, hydrodynamic injection of SB or PB transposon constructs carrying a library of sgRNAs (along with Cas9) allows for multiplex mutagenesis and cancer induction [79, 80]. As the transposon gives stable integration of the CRISPR constructs into the host genome, the sgRNA cassette can be subsequently identified as a readout. Transient electroporation of sgRNAs can also be performed [81, 82], with fewer off-target effects due to lack of stable Cas9 and sgRNA expression. These methods of transfection also allow for delivery of multiple CRISPR plasmids into each cell, enabling random multiplexed mutagenesis [79].

The use of viral vectors has extended the range of target tissues amenable to CRISPR screens. Integrating viruses such as lentivirus accommodate a relatively large DNA cargo (~ 8–10 Kb) and their ability to self-integrate into the genome. For example, lentiviral sgRNA/Cre pools targeting TSGs were administered intratracheally into conditional Cas9 and *Kras*^*G12D*^ transgenic mice to generate lung tumors [83]. Non-integrating viral vectors, in particular single-stranded adeno-associated viruses (ssAAVs), albeit presenting a reduced carrying capacity (~ 4.7 Kb), have higher transduction rates compared with lentivirus and low immunogenicity [84]. Given the limited cargo capacities of viral vectors, it is important to maximize the usefulness of the plasmids being delivered; as such, Cas9 knock-in mice, including conditional ones with tissue-specific Cas9 expression, obviate the need to deliver Cas9 with a viral vector and also help prevent immune clearance of Cas9-expressing cells when Cas9 is delivered in this way [77]. Alternatively, smaller versions of Cas9, such as *Staphylococcus aureus* Cas9, can be delivered together with sgRNAs using viral vectors [85]. Finally, nanoparticles, including those based on lipid or gold, have a large cargo capacity and can be used for delivery of Cas9 and sgRNAs, typically with low immunogenicity [86–90]; early results show efficient genome editing in vivo with such nanoparticle delivery techniques.

In sum, CRISPR screens can now be deployed to examine many aspects of cancer genetics in an in vivo setting, with newer developments promising to provide unprecedented biological insight.

## Comparison of transposon and CRISPR cancer screens

**Fig. 2:**
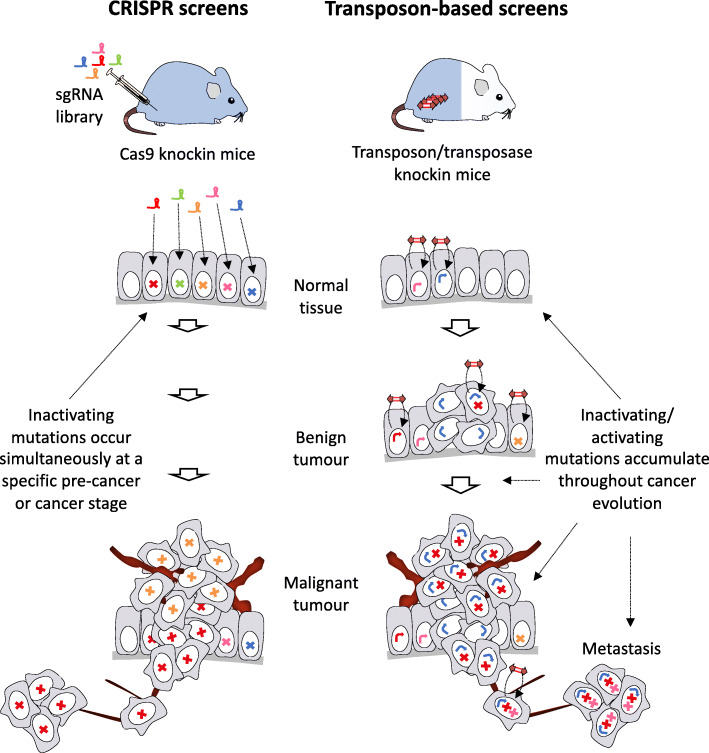
CRISPR and transposon mutagenesis screens enable cancer gene identification in vivo. *Left*: a typical pooled, sgRNA CRISPR library is transduced into Cas9-knockin mice, leading to the loss of a TSG in each cell, driving oncogenesis. Multiplexed delivery of sgRNA constructs can alternatively be achieved leading to simultaneous editing of multiple TSGs (not represented). *Right*: mice harboring transposons and transposase develop spontaneous tumors due to transposon-induced activating and inactivating mutations. Both oncogenes and TSGs can be identified by deep sequencing of transposon hits at different stages of tumor progression, revealing genetic routes of cancer evolution. Symbol notation: cross = inactivating mutation; bent arrow = activating mutation; different colors denote mutations in different cancer genes

Transposon and CRISPR screens are complementary tools in cancer. In contrast with transposon mutagenesis where there is random genome-wide screening for cancer genes, CRISPR offers additional versatility by allowing for focused genetic screens in cancer where genes of potential interest are pre-specified (Fig. 2). CRISPR-induced mutations are set after the time of sgRNA library delivery, whereas with transposon mutagenesis mutations continue to occur during a screen due to transposition. This ability to accumulate mutations throughout tumor evolution mimics human cancer progression and allows for the interrogation of genes at different disease stages. For example, *EGFR*-initiated gliomas were noted to have high genomic instability including large chromosomal deletions, presumably reflecting the need for additional driver events: PB enabled pinpointing of these collaborating cancer genes [91].

A common feature of CRISPR screens is that they are purely loss-of-function in the context of Cas9, or purely gain-of-function when CRISPRa is employed (Table 1). This differs from most transposon mutagenesis screens which use SB or PB transposons that can either activate or inactivate genes in the same tumors. Although having both gain- and loss-of-function capability in the same screen is advantageous, computational interpretation of which genetic hits are oncogenes or TSGs can be challenging, particularly for CIS with few insertions. Consequently, exclusively inactivating transposon platforms have been designed that simplify downstream interpretation with all hits being putative TSGs; this has been informative in identifying new B cell lymphoma drivers with PB [92]. We anticipate the development of mouse lines carrying transposons with purely activating sequences will facilitate genome-wide screens focused on characterizing oncogenes.

**Table 1:** Comparison of transposon mutagenesis and CRISPR screens in vivo

Feature	CRISPR	Transposon
Cancer model quality	Transplantation models: limited by lack of immune system. Autochthonous models: high quality.	High-quality given autochthonous nature.
Tissue delivery of components	Can be technically challenging. Options include: - Transfection, with or without PB/SB: allows multiplexing; limited to certain organs. - Lentivirus or AAV: wide range of tissues are accessible; relatively limited cargo capacity. - Nanoparticles: high cargo capacity, low immunogenicity.	Constitutive and conditional transposase mouse knock-in alleles are available. No further delivery of exogenous components is required.
Efficiency of mutations	High efficiency in vitro (for transplantation model). In vivo mutagenesis has typically lower efficiency than in vitro*.*	High efficiency of gene disruption in vivo; gene activation efficiency is variable. Therefore, TSGs are more identifiable than oncogenes^1^.
Types of mutations	Disruptive (indels)/knockout. Transcriptional activation/repression. Translocations/deletions. Point mutations.	Disruption or activation of expression. Drop-out screens are not possible.
Alleles targeted	Can induce mutations in both alleles of diploid cells	Usually only one allele is mutated in diploid cells.
Unwanted genetic effects	Low off-target effects. On-target unintended effects may occur.	Local hopping Footprint mutations with SB.
Genome coverage in screen	Focused or genome-wide libraries. High library coverage can be challenging to achieve in vivo*.*	Whole-genome including non-coding regions. PB favors open chromatin. SB and PB have differing integration preferences.
Time to conduct screen	Relatively short: direct injection of CRISPR components into tissues is required.	Relatively long: compound mutant mice (typically three or four alleles) need generating.
Costs of screen	Relatively low.	High costs due to long mouse breeding times.

^1^Gene disruption is more likely given that the position and orientation of the transposon relative to the inserted gene are less critical than for gene activation

Undesired genetic effects also differ between transposons and CRISPR; although CRISPR-Cas9 has low off-target effects (unintended modifications at other sites in the genome) in comparison with prior RNAi screens [93–95], these warrant consideration in screens, justifying the use of multiple sgRNAs per gene and of new computational tools to improve sgRNA design [96–102]. Unintended on-target effects have also been noted with Cas9 [103]. Cas9 expression has been found to activate the p53 pathway and select for *TP53*-inactivating mutations, presenting a potential caveat of Cas9 tumor models [104]. Aside from footprint mutations that occur with SB and local hopping with both transposon types, SB and PB have integration preferences that may present false positives in cancer screens, requiring downstream validation studies to exclude these. Regarding the nature of mutations, with CRISPR, both alleles of a diploid cell can become mutated, whereas transposons typically cause heterozygous mutations; this can be advantageous meaning transposon screens are able to detect haploinsufficient tumor suppressors, particularly in the context of a *Bloom*-deficient background which favors loss of heterozygosity of inactivating mutations by increasing mitotic recombination [105, 106]. Moreover, compared with transposon mutagenesis, CRISPR is able to recapitulate a larger repertoire of genetic changes seen in human cancer, including point mutations [107–109], and allows for negative-selection (“drop-out”) screens.

CRISPR in vivo screens have associated challenges. It is often difficult to achieve high library coverage for genome-wide in vivo screens given that very many cells would need to be transduced with sgRNAs to maintain library representation, meaning that investigators typically choose to use focused sgRNA libraries for such screens. Moreover, the multiplicity of infection is difficult to control, delivery efficiency rates can be low, the accessibility of the target tissue to CRISPR libraries may be challenging, and the host immune response to delivery vectors may render the complete clearance of the targeted cells. Transposon mutagenesis screens lack these difficulties, allowing the study of virtually any cancer type with genome-wide coverage, without such immune responses, although transposon screens are more time-consuming given the need for crossing multiple alleles.

**Fig. 3:**
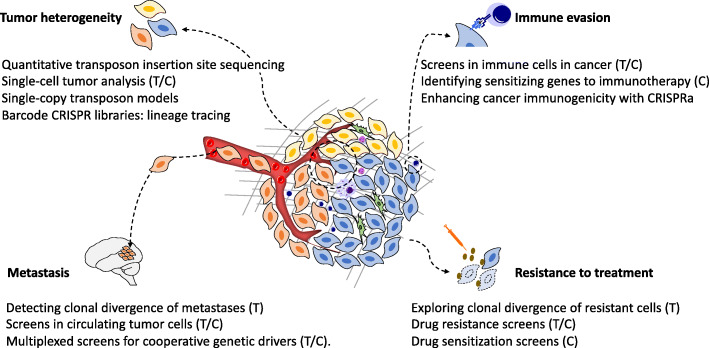
Applications of CRISPR and transposon mutagenesis for in vivo cancer screens. Cancer is typically genetically and phenotypically heterogeneous, and immune cells in the microenvironment play a pivotal role in oncogenesis. Key areas for investigation of cancer by in vivo screens include genetic mechanisms of tumor heterogeneity, metastasis, immune evasion, and resistance to treatment. “T” = transposon mutagenesis; “C” = CRISPR

We next discuss how CRISPR and transposons screens can be harnessed for understanding cancer heterogeneity, metastasis, and resistance to treatment (Fig. 3).

## Truncal drivers and intratumor heterogeneity

Intratumor heterogeneity, resulting from branching tumor evolution, complicates efforts to decipher important drivers of cancers and therefore to develop effective precision therapies [110–112]. Within a tumor, there is phenotypic variation in cancer cell and immune cell types, in addition to different subclonal mutational and transcriptional signatures that influence these phenotypes [113]. An attractive therapeutic approach is to target the “truncal drivers,” those genetic changes that are present at the trunk of the cancer evolutionary tree and which sustain growth of multiple subclones rather than more minor changes present only in small subpopulations of tumor cells [114, 115]. These truncal mutations will be present in the majority of cells within a tumor, providing potentially attractive neo-antigens for immunotherapy and targets for inhibiting oncogenic signaling [116].

Transposon mutagenesis is a powerful tool for defining truncal driver genes, given that such genes will have transposon integrations with the highest sequence read counts (suggesting high clonality), making them readily discernible. This has been exploited by multiple studies with SB. For example, a hepatocellular carcinoma (HCC) SB screen in a chronic hepatitis B autochthonous model yielded 21 truncal drivers compared with 2860 candidate late-stage drivers; 1199 genes were related to hepatocellular metabolism, including truncal drivers such as *Pten*, *Adk*, and *Zbtb20*, suggesting a prominent role for aberrant metabolism in HCC [117]. Takeda et al. utilized different predisposing mutations (*Apc*^*min*^, *Kras*^*G12D*^, *Trp53*^*R172H*^, and *Smad4*^*+/−*^) in SB screens to investigate the genetic evolution of gastrointestinal tumors in autochthonous models [118, 119]. These screens yielded 142 candidate trunk driver genes. *Apc,* an antagonist of the Wnt pathway, was the most frequently mutated trunk gene in *Apc-, Kras*-, and *Trp53*-mutant tumors, consistent with *APC* loss being the main mechanism for initiation of human CRC. By contrast, *Smad4*-mutant tumors presented frequent trunk insertions in the wild-type *Smad4* allele along with mutually exclusive activating insertions in Wnt receptor agonist *Rspo1* and *Rspo2,* highlighting different mechanisms for WNT signaling activation operating in this genetic context.

These studies have proved successful for discriminating truncal driver genes amongst the many genes that are hit by transposition. However, transposon insertions were identified based on PCR amplification of transposon-containing fragments obtained from enzymatic digestion of tumor DNA, which results in amplification biases due to the uneven genomic distribution of restriction enzyme recognition sites. Alternatively, acoustic shearing of tumor DNA mitigates this bias by fragmenting DNA at random points, allowing for a semiquantitative analysis of the relative clonality of individual insertions within heterogeneous tumors [48, 92, 120, 121]. Moreover, Mann et al. have developed SBCapSeq, a method to identify SB transposon insertion sites from single tumor cells [122]. Application of SBCapSeq to an SB model of myeloid leukemia enabled single-cell resolution of transposon hits, identifying cooperating trunk drivers *Erg* and *Ghr*, and *Notch1* and *Ets1* in major dominant subclones. This approach can conceivably be adapted to PB.

Most transposon mouse lines have multiple copies of the transposon mobilizing in each cell, presenting a challenge for identifying the key molecular players amongst tumor subclones. To help resolve this complexity, de la Rosa et al. employed a single-copy of the SB transposon and coupled its mobilization to the inactivation of one *Pten* allele within each cell on a *Bloom*-deficient background. The analysis of prostate, breast, and skin tumors arising de novo from these mice identified novel TSGs cooperating with *Pten* in these cancer types, of which *Zbtb20*, *Celf2*, *Pard3*, *Akap13*, and *Wac* were further validated as prostate cancer drivers [105, 123]. This model had advantages compared with multiple-copy transposon models: having only one copy of the transposon per cell reduced passenger mutations, and transposition was coupled to *Pten* inactivation within each genome, which may increase the sensitivity of the screen for identifying *Pten*-cooperative cancer genes. Single-copy models can be further exploited to interrogate intratumor heterogeneity by dissecting the clonality of transposon insertions even from bulk sequence analysis.

CRISPR in vivo screens serve as an essential tool for investigating how tumor subclones interact with one another in controlled experimental settings. The AAV-CRISPR platform facilitates functional interrogation of intratumoral heterogeneity, as demonstrated by recent examples. Stereotaxic delivery into the brain of conditional Cas9 mice of an ssAAV sgRNA library, targeting *Trp53* together with recurrently mutated candidate cancer genes, induced tumors in an autochthonous setting that recapitulate human glioblastoma [84]. The same AAV vectors were also injected intravenously for the study of liver cancer [14]. Given the higher transduction efficiency of ssAAV, tumors had multiple mutations, and co-operative driver events could be identified by determining patterns of co-occurring CRISPR-induced mutations. These brain and liver cancers were composed of competing subclones, mimicking the genetic complexity of intratumoral heterogeneity seen in human cancers that is often not recapitulated in other genetically engineered mouse models [32]. The clonal dynamics of these tumors are simpler to interpret because of the relatively few mutated genes observed compared with human cancer, and that the genes are pre-specified by the library shifting focus to a core set of genes-of-interest.

Single-cell sequencing technologies are rapidly evolving. We anticipate that single-cell RNA-sequencing (scRNA-seq) will allow characterization of cancer genotype-phenotype links at high resolution for understanding tumor heterogeneity and truncal driver effects in transposon cancer models. Advances in computational tools have improved the detection of fusion transcripts, which are produced by transposon insertions: these transcripts are initiated by the transposon and splicing to downstream exons, or they are initiated by the endogenous cell promoter and splicing to the transposon [124, 125]. Detection of these transcripts as part of transcriptomic analysis enables comparison of transposon hits with tumor gene expression profiles to link genotypes with cancer phenotypes, although this has yet to be performed for single cells.

To pinpoint cells derived from a common ancestor for the purposes of lineage tracing, DNA barcodes can be integrated in CRISPR vectors to deconvolute bulk tumor populations into individual clones with specific mutations through deep sequencing [83, 126, 127]. Recent integration of pooled CRISPR screens with single-cell RNA sequencing has allowed linking of each gene knockout with its effect on the cell’s transcriptome, disentangling cellular heterogeneity at ultra-high resolution [128]. Several methods (Perturb-Seq, CRISP-seq, or CROP-seq) have been engineered to accomplish this, all relying on the indirect index of the sgRNAs with poly-adenylated unique molecular identifiers that can be detected by mRNA-seq [129–131]. Newer innovations have been introduced which allow multiple distinct sgRNAs expressed in each cell to be directly sequenced alongside their transcriptomes (Direct-capture Perturb-seq) [132]. This technology circumvents the limitations associated with the indirect barcoding of sgRNAs of previous methods (including poor scalability, dependence on specialized vector systems, and high cost) and allows high-throughput investigations of genetic interactions. These methods have been employed in cell lines, yet application to cancer cells in de novo tumors is eagerly anticipated. Such innovations are likely to yield deeper insights into intra-tumor heterogeneity from CRISPR screens.

## Genetic determinants of metastasis

The development of metastases at sites distant from the primary tumor is a hallmark of cancer [133], portends major treatment challenges, and is a leading cause of cancer-related deaths. Metastasis depends on complex genetic interactions that are still poorly understood, particularly in comparison with underlying primary tumors. Metastasis requires a sequence of events that includes invasion of cancer cells into surrounding tissues, seeding of these cells into the blood (and lymphatic) circulation, and deposition of these cells into distant tissues to form micro-metastases and subsequently full-blown metastases reaching threshold for clinical detection [134]. In vivo cancer screens are paving the way for understanding these interactions between driver genes in metastatic cancer evolution.

An SB screen designed to elucidate cancer genes in medulloblastoma (a brain cancer) utilized heterozygous *Ptch1* and heterozygous *Trp53* mouse backgrounds to mimic the human cancer in an autochthonous context. These screens discovered high genetic divergence between primary medulloblastomas and their metastases, with the metastases containing driver events from a restricted subclone of the primary tumor. Clonal insertions were observed in oncogenes *Notch2* and *Tert* in primary tumors but not metastases, whereas insertions were present in PI3K pathway genes such as *Pten*, *Akt2*, and *Pik3r1* exclusively in metastases. These results could imply that targeted therapies against truncal drivers of primary medulloblastomas will not remove the metastases [135]. An osteosarcoma SB screen in mice with and without *Trp53* somatic loss reported 232 CIS genes associated with de novo primary cancer development and 43 CIS genes associated with metastases (for example, *Pten*, *Nf2*, and *Snap23*). Most metastases shared several genetic insertions with matching primary osteosarcomas, suggesting shared clonal evolution of primary and metastatic tumors during oncogenesis; some metastases however shared few insertions with the primary tumor, implying an early metastatic event in those cases and highlighting heterogeneity in tumor evolutionary forces [136]. Transposon screens can also help define molecular determinants of other processes needed for metastasis such as the epithelial-mesenchymal transition (EMT) [134, 137].

In comparison with transposon mutagenesis, where transposons continue to mobilize even after cancer genes are “hit,” CRISPR mutations are generated right at the beginning of a cancer screen. This means that for transposon screens, additional driver genes can be pinpointed that yield metastases that may not be present in the primary tumors. With CRISPR, the initial group of pre-specified sgRNAs in the library sets the limit for driver mutations (although selection pressures continue to direct tumor evolution), allowing a screen to focus specifically on one stage of cancer evolution, such as metastasis. Repeated delivery of CRISPR libraries may allow interrogation of multiple disease stages, although it is likely to be technically challenging to target the same cells. One of the first CRISPR in vivo cancer screens introduced a genome-wide loss-of-function sgRNA library into a previously non-metastatic mouse cancer cell line expressing Cas9*,* then subcutaneously transplanted these cells into immunocompromised mice, and monitored them for lung metastases [138]. Genetic sequencing for the sgRNAs in metastases enabled identification of genes functionally promoting cancer dissemination.

Circulating tumor cells (CTCs) in the bloodstream are recognized as an intermediate stage of metastasis, with a fraction of these cells being capable of colonizing distant organs and initiating metastases [139]. CRISPR screens have provided a new opportunity for studying the biology of CTCs. A recent demonstration of this comes from a genome-wide gain-of-function screen in a mouse transplant model using the CRISPRa system in CTCs derived from breast cancer patients. Screen hits from metastases were enriched for ribosomal proteins and regulators of translation. In particular, overexpression of one candidate, *RPL15*, led to increased metastasis in mice [140]. These gain-of-function genetic targets are likely to be more susceptible to drug targeting than those that are loss-of-function, which typically require indirect approaches for therapeutic targeting.

Given that metastases generally require additional driver mutations compared with early tumor stages [141], a promising novel approach for deciphering metastasis drivers is by the use of an alternative version of Cas, CRISPR-associated endonuclease in *Prevotella* and *Francisella* 1 (Cpf1, also called Cas12a). Cas12a enables genome editing at multiple loci (up to 25 so far) to occur simultaneously using a single CRISPR array [142, 143]. A screen used this system to generate double knockouts in a cancer cell line, with a library of candidate metastasis-causing genes [144]. Transplantation of these cells into the skin of immunocompromised mice led to primary tumors and lung metastases, sequencing of which revealed gene pairs clonally selected for in metastatic development such as *Nf2* and *Trim72* double knockouts. This platform will thus shed further light into the cooperative genetic partners that promote cancer dissemination. Given that CRISPR-mediated mutations rely on double-strand breaks, having many of which may lead genome instability and possible cell death, further exploration of RNA-guided ribonucleases is warranted as a potential option for multiplexed screens [54].

## Genes influencing response to treatment

A key factor affecting survival of cancer patients is that cancers develop resistance to therapeutic drugs, driven by novel alterations in cancer cells that selectively provide a survival advantage in the presence of drugs. Clonal dynamics ensure that cancers evolve in a way that makes them resistant to those drugs (such as EGFR inhibitors for *EGFR*-mutant colorectal cancer [145]), demanding the use of newer agents for recurrent tumors. The genome of these “end-stage” cancers is typically complex given the wealth of genetic and epigenetic alterations accumulated during their life history (most of which are of uncertain significance). This makes discerning genes driving therapeutic resistance all the more challenging. Functional genomic screens are increasingly being adapted to solve these problems, including drivers of cancer relapse after treatment, and of sensitivity and resistance to drugs.

An illustrative approach comes from an SB screen for genes giving resistance to therapy in a “humanized” autochthonous mouse model of heterozygous *Ptch1* medulloblastoma [146]. The SB-primary tumors were microsurgically removed and the mice treated with radiotherapy, reflecting the standard of care in human patients. Genetic sequence analysis of the recurrent tumors revealed different CIS genes between primary and relapsed medulloblastomas. For example, clonal insertions in *Arid1b* and *Tcf4* were observed in metastases but not in primary tumors. Moreover, the dominant clone of relapsed medulloblastomas arose partly through clonal selection (imposed by surgery and radiotherapy) of a minor subclone present in the primary tumor. These findings suggest that treatments aimed at truncal mutations in the primary tumor are unlikely to cure patients if they are not present at relapse. Similar screens have been conducted with SB for genes driving resistance to a BRAF inhibitor in *Braf*^*V600E*^-driven melanoma in an autochthonous model [147] and with PB revealing convergent mutations in *Trp53* as a resistance mechanism of allografted tumors to MDM2 inhibitors [148]. Future transposon mutagenesis screens to identify the molecular players driving tumor recurrence in other cancer treatment contexts, including immunotherapies, are warranted.

CRISPR provides a powerful alternative for studying genes driving either resistance or sensitivity to therapy in vivo. Genes mediating sensitivity or resistance to therapy can often be identified in the same CRISPR screen by analyzing sgRNAs that are depleted or enriched respectively, maximizing gene discovery. Various computational approaches have been developed to interpret biologically significant genes in these contexts [149, 150]. A study employing dCas9-trancriptional activators to perform a gain-of-function screen in a syngeneic immunocompetent transplant model discovered *Chek2* transcriptional activation leads to increased sensitivity of B-cell acute lymphoblastic leukemia (B-ALL) to the chemotherapy temozolomide, slowing down bone marrow relapse [151]. Also, the druggable oncogene *KPNB1* was identified in a loss-of-function CRISPR screen in a xenograft model; KPNB1 inhibition reduced epithelial ovarian cancer growth in vivo [152]. More recently, Li and colleagues used a multiplexed lentiviral approach to deliver sgRNAs against tumor suppressors in a *Kras*-driven autochthonous lung cancer mouse model; tumors were treated with a panel of drugs, and the screen identified *Keap1* mutations as a resistance mechanism to carboplatin treatment [153], highlighting that cancer cell sensitivity to drugs can be genotype-specific.

It is now recognized that small-molecule inhibitor drug screens suffer from off-target toxicity: some drugs kill cells even when their putative genetic targets are mutated by CRISPR-Cas9 [154, 155]. Although this has only been shown for a selected set of drugs, this may partially explain why pre-clinical oncology drugs often fail in cancer patients. In comparison, CRISPR-Cas9 has low off-target effects [98, 156], making drug target identification more specific.

## The immune environment and immunotherapy genetic targets

The immune system is important for suppressing most potentially cancerous cells from ever forming tumors by recognizing and destroying them at early stages. Cancers that arise have therefore bypassed this critical immune surveillance, likely because immunogenic clones have been removed leaving weakly immunogenic variants to progress, so called immunoediting [133]. This hypothesis has been supported by cancer transplantation experiments in mice [157] and more recently from human cancer genome sequence data [158]. Although the mechanisms of cancer immune escape are far from fully understood, a prominent example is through T cell suppression by cancer cell upregulation of PD-L1 [159], which is exploited clinically in several cancers with major success [160].

A PB screen in T cells discovered *PDCD1*, which encodes PD-1, as a key suppressor of oncogenic T cell signaling that prevents development of T cell lymphoma in an autochthonous mouse model; human lymphomas contain deletions in this gene in up to 30% of cases, confirming clinical relevance [161]. Although PD-1 is well-established as an immunotherapeutic target, this study showed how it also acts as a TSG by increasing levels of PTEN. Accordingly, loss of *PDCD1* leads to increased PI3K-AKT signaling that contributes to T cell lymphoma development.

Moreover, efforts are underway to define further targets on T cells. To define novel membrane targets on CD8+ T cells that enhance killing of glioblastoma cells, Ye et al. developed a hybrid genetic screening platform where an SB transposon carrying an sgRNA library is nested in an ssAAV. This enables efficient genomic integration of the sgRNA cassette for the screen readout. The AAV-SB-CRISPR library was transduced into Cas9-expressing mouse CD8+ T cells, which were transplanted into mouse brain tumors; validation of the screen hits was performed by adoptive transfer of CD8+ T cells with *Pdia3*, *Mgat5*, *Emp1*, or *Lag3* knockouts, which improved survival of GBM-bearing mice [162].

Other efforts are focusing on identifying targets on cancer cells that more effectively engage T cells. A CRISPR screen set out to identify genes which can sensitize melanoma to PD-1 inhibition or make it more resistant [13]. A pooled sgRNA library targeting over 2000 genes (including plasma membrane proteins and those for antigen presentation) was transduced into murine melanoma cells expressing Cas9, which were then transplanted into mice. Knockout of PTPN2, which encodes for a protein tyrosine phosphatase, unexpectedly led to improved efficacy of immunotherapy by increasing interferon-gamma-mediated effects on antigen presentation. Another CRISPR screen used an sgRNA library focusing on epigenetic regulators in a syngeneic lung cancer transplant model, identifying *Asf1a* sgRNAs as being significantly depleted, and validation studies confirmed that *Asf1a* knockout synergized with anti-PD1 therapy to slow tumor progression by promoting T cell activation [15]. Such screening approaches can feasibly be tailored to discover new therapeutic targets for immunotherapy in other cancer contexts [163].

As genetic and epigenetic alterations accumulate during tumor evolution, they have potential to generate an anti-tumor immune response and rejection if these tumor-associated antigens (TAA) are adequately presented [164]. However, most such TAAs are inadequately presented and there is usually neoantigen loss [165], allowing cancer to evolve whilst evading the immune system. Recent work has leveraged CRISPRa for multiplexed activation of TAAs to enhance anti-tumor immunity. Injection of an sgRNA library targeting known mutated genes into mouse cancer cells expressing dCas9-transcriptional activators led to increased expression and subsequent presentation of TAAs [166]. Tumor regression occurred because of increased T cell infiltration and tumor cell destruction in this transplant model. These screens illustrate the therapeutic potential of CRISPRa to augment anti-tumor immunity based on detailed knowledge of an individual patient’s cancer gene profile.

In summary, functional genomic screens are generating unprecedented insights into immune regulators of oncogenesis and beginning to pinpoint new immunotherapy targets.

## Future directions for transposon and CRISPR screens

The large-scale data being provided through human oncogenomic studies and in vitro screens will require complementary advances in autochthonous mouse models and in vivo screens to provide deeper insights into cancer biology. Here, we highlight areas for future development in functional genomic screens in cancer.

Although most screens have focused on protein-coding genes, these account for only 2% of the human genome, demanding exploration of non-coding genomic regions [167–169], of which enhancers (genetic elements regulating transcription of distant genes by chromatin looping) make up a large part and can contribute to oncogenesis [170]. CRISPR screens can be used to characterize functional enhancers, at least in vitro, by targeting Cas9 to transcription factor binding sites in enhancer regions [171]. Application of such technology in vivo and for therapeutic resistance investigations is worthwhile future lines of study.

Critical developments needed for functional interrogation of cancer evolution are precision-modeling of cancer genetic and epigenetic alterations, multiplexing of cancer drivers, and technologies for controlling the temporal order of mutations. The use of dCas9-transcriptional activator mice will enable CRISPR screens to identify oncogenes in vivo [172]*,* and we anticipate the development of PB or SB transposons with purely activating sequences could allow this to be performed with insertional mutagenesis. Given that the type of alteration may influence their oncogenic potential, mice expressing CRISPR base-editors may be developed to screen multiple specific point mutations in autochthonous settings, and mice engineered to express dCas9 fused to DNA methyltransferases may enable screens for epigenetic drivers. It is conceivable that more complex genetic changes such as chromosomal rearrangements may be amenable to high-throughput in vivo screens with advances in CRISPR technology. With the rapid progress in multiplexed CRISPR technologies [173], expression of many gRNAs per cell will allow us to discover synergistic gene interactions hidden within complex cancer genetic networks. Moreover, we envision the use of several Cas enzymes in tandem [174] may enable CRISPR screens to detect both oncogenes and TSGs in vivo. Inducible transposon systems can be engineered to switch on transposition at particular times during development or adulthood to interrogate the role of timing of mutations on cancer development [175]. Similarly, inducible systems regulating the expression of Cas9 by external trigger signals (i.e., small molecules, light, or temperature) have been developed [176]. Also, engineering of CRISPR constructs with multiple sgRNAs whose expression can be switched from one sgRNA to another by inducible expression of Cre is a step in this direction [177], as are in vivo inducible lineage tracing tools that allow tracking of clonal origins of cell populations in high-resolution [178].

To overcome some limitations with CRISPR library delivery to tissues, engineered mosaic mice harboring germline floxed sgRNAs linked together in tandem are in development, with one sgRNA expressed per cell following Cre-mediated recombination [179]. Double-stranded AAVs (dsAAVs), which result from a mutation on one of the viral inverted terminal repeats (ITR), have provided further improvements in viral transduction efficiency and CRISPR-mediated genome editing, although at the cost of a reduced package capacity (~ 2.5 kb) compared to ssAAVs [180]. The integration of SB or PB transposons carrying CRISPR libraries within the ITRs of AAVs allows the genomic integration of the sgRNA cassettes for their easy detection [162], holding promise for large-scale cancer CRISPR screens.

With the advent of single-cell sequencing, intratumor heterogeneity can be interrogated by searching for combinations of sgRNAs or gene-transposon fusion transcripts respectively across thousands of cells in a tumor and matching these with phenotypes inferred from cells’ transcriptomes. Refinements in technology may further enable screens to be performed directly in immune cells in vivo to identify novel regulators of immune activation in cancer as potential immunotherapy targets. Ultimately, screens that are tailored to patient-specific genetic profiles will yield personalized cancer driver information that can inform precision therapeutics. As an example, human tissue organoids (engineered to express Cas9) can be transduced with a CRISPR library of a patient’s mutated genes and transplanted into mice to determine the effects of individual mutations on clonal growth dynamics [181]. We envision that development of a patient “avatar” approach [11], employing personalized gene libraries for screens, will refine therapeutic testing strategies to more accurately model the influence of drugs for individual patients in the clinic.

The power of large-scale transposon mutagenesis and CRISPR screens, made more relevant with recent and future developments, will equip us with the tools needed for gaining unprecedented insights into functional drivers of cancer evolution and therapeutic targets for clinical translation.

## Supplementary information

**Additional file 1.** Review history.
