# Supplementary material for: CRISPR and transposon in vivo screens for cancer drivers and therapeutic targets
Source: Genome Biol. 2020 Aug 19;21:204. doi: 10.1186/s13059-020-02118-9 (PMC7437018; doi:10.1186/s13059-020-02118-9)
Supplement: Supplementary file 1 — Additional file 1. Review history. [file 13059_2020_2118_MOESM1_ESM.docx]

Review History

**First round of review**

**Reviewer 1**

**Comments to author:**

Though the authors focus on transposon and CRISPR screens, it is worthwhile mentioning RNAi during the introduction and throughout the manuscript. also in the future plans, the advanteges of RNAi screens can be highlighted in comparison (for example the flexibility of knockdowns versus knockouts and the high off target effects).

The authors mention the non-coding genome (in future direction paragraph), but cite only references concerning non-coding RNAs (159, 160, 161). However, the non-coding genome is to a large extent composed of regulatory DNA elements (such as transcriptional enhancers) which can be highly relevant to cancer progression and metastasis. Their targeting and functional screening was shown to be possible in vitro (e.g. Korkmaz et al., 2016 Nature Biotechnology). Whether they can be used in drug resistance and in vivo is still unclear, but an interesting future relevant direction. It seems that this can be mentioned in the introduction, and also as future in vivo research lines in the discussion.

On page 4 4th paragraph, the authors mention efficient in vivo delivery of sgRNAs by several methods. This can be detailed more, including a list of methods and their comparison to SB and PB. A table can be considered too.

**Reviewer 2**

**Comments to author:**

In this review, Noorani et al. discusses major technological breakthroughs utilizing transposon and CRISPR screens. In the context of these approaches, this review discusses how genetic screening can provide insight into cancer evolution, intratumoral heterogeneity, metastasis, and immune evasion. Overall, the is a well written and thoughtful review that provides a comprehensive and up-to-date discussion on current technology for transposon and CRISPR screening. The review also does an excellent job comparing the strengths and weaknesses of both screening methods. The discussion of future directions does an excellent job defining the goals of the field for years to come.

Some suggestions for the authors to consider:

1)	On page 7, the paragraph on integration with scRNA-seq seems a little out of place, although I am not certain where it would fit better. Perhaps closer to the end of that section around the barcoding and Perturb-seq section.

2)	On page 8, in the paragraph on CRISPR-UMI, Perturb-seq etc I think that it is important to point out that these methods have been employed in cell lines but not yet on cancer cells in de novo tumors

3)	Within the CRIPSR toolbox that has been employed in vivo that authors do not mention much about base-editing or HDR to change individual bases. Base editing is shown in Fig 1 but not really discussed (except in future direction). HDR is currently pretty low efficiency in vivo, but it has been used and probably has some utility (especially in the future if efficiency can be increased). Not sure if these methods warrant a few more sentences or not.

4)	The authors do a good job at the beginning highlighting that there is an important difference between cell lines transplant and autochthonous tumor models. It might be nice for the authors to go through the manuscript and make sure that this is clear for most of the examples that they highlight. I think that this is an important distinction.

5)	Suggesting some caution around genome scale in vivo screen could we warranted. The math around maintain representation in these screens is not always kept in mind even by the authors of those papers. I think that the limitation on the number of genes that can be interrogated in most in vivo CRISPR screens in a negative relative to transposon screens.

6)	The section on CRISPR approaches in drug responses to treatments is pretty minimal. I imagine that this is because that data using these approaches in vivo have been limited. There are 2 manuscripts on Biorxiv where CRIPSR/CAs9 screens have been used to create diverse lung tumor genotypes followed by drug treatments (https://www.biorxiv.org/content/10.1101/2020.04.13.036921v1 and https://www.biorxiv.org/content/10.1101/2020.01.28.923912v1). I am not sure whether these are worth citing or not.

7)	I don’t entirely agree with the statement referencing refs 146, 147. Those drugs were highly selected etc.

8)	The logic to outline a dual sgRNA screen in Figure 2 is unclear. I sort of field like a single sgRNA screen in more “typical”

This is really a very thoughtful and interesting review.

**Authors Response**

We would like to sincerely thank the reviewers of our manuscript for their insightful comments. We have given plenty of consideration to these comments and have revised our manuscript to address these comments as best as possible. As a result, we feel the manuscript has considerably improved and we hope will provide a useful resource for the scientific community employing CRISPR and transposon screens *in vivo* for cancer gene discovery. Here, we provide a point-by-point response to the reviewers.

Reviewer 1:

Though the authors focus on transposon and CRISPR screens, it is worthwhile mentioning RNAi during the introduction and throughout the manuscript. also in the future plans, the advantages of RNAi screens can be highlighted in comparison (for example the flexibility of knockdowns versus knockouts and the high off target effects).

- Thank you. RNAi screens have provided a hugely important contribution to the cancer field. We have now mentioned this in the Introduction and provided a brief comparison with CRISPR screens, as well as references for more in depth discussion given the focus of this review is on CRISPR and transposon screens *in vivo*.

The authors mention the non-coding genome (in future direction paragraph), but cite only references concerning non-coding RNAs (159, 160, 161). However, the non-coding genome is to a large extent composed of regulatory DNA elements (such as transcriptional enhancers) which can be highly relevant to cancer progression and metastasis. Their targeting and functional screening was shown to be possible in vitro (e.g. Korkmaz et al., 2016 Nature Biotechnology). Whether they can be used in drug resistance and in vivo is still unclear, but an interesting future relevant direction. It seems that this can be mentioned in the introduction, and also as future in vivo research lines in the discussion.

- Many thanks. The role of enhancers in the non-coding genome is being increasingly recognized to play an important role in cancer. We have now hinted at this in the Introduction, cited the reference of Korkmaz et al 2016, and given consideration to this area in the Future Directions.

On page 4 4th paragraph, the authors mention efficient in vivo delivery of sgRNAs by several methods. This can be detailed more, including a list of methods and their comparison to SB and PB. A table can be considered too.

- Thank you. We have now provided extra detail on the various methods of *in vivo* sgRNA delivery in this paragraph, including transfection, lentiviral and adenoviral methods, and nanoparticles. Additional detail has also been added about these methods in the main table (Table 1).

Reviewer 2:

In this review, Noorani et al. discusses major technological breakthroughs utilizing transposon and CRISPR screens. In the context of these approaches, this review discusses how genetic screening can provide insight into cancer evolution, intratumoral heterogeneity, metastasis, and immune evasion. Overall, the is a well written and thoughtful review that provides a comprehensive and up-to-date discussion on current technology for transposon and CRISPR screening. The review also does an excellent job comparing the strengths and weaknesses of both screening methods. The discussion of future directions does an excellent job defining the goals of the field for years to come.

Some suggestions for the authors to consider:

1. On page 7, the paragraph on integration with scRNA-seq seems a little out of place, although I am not certain where it would fit better. Perhaps closer to the end of that section around the barcoding and Perturb-seq section.

- Thank you. We agree that this paragraph fits slightly better just before the barcoding section, where there is more single cell work discussed. This has now been amended.

2. On page 8, in the paragraph on CRISPR-UMI, Perturb-seq etc I think that it is important to point out that these methods have been employed in cell lines but not yet on cancer cells in de novo tumors.

- Thank you. This is an important point regarding the differentiation between *in vitro* and *in vivo* work, which we have now pointed out.

3. Within the CRIPSR toolbox that has been employed in vivo that authors do not mention much about base-editing or HDR to change individual bases. Base editing is shown in Fig 1 but not really discussed (except in future direction). HDR is currently pretty low efficiency in vivo, but it has been used and probably has some utility (especially in the future if efficiency can be increased). Not sure if these methods warrant a few more sentences or not.

- Many thanks. CRISPR base-editing has been important addition to the CRISPR toolkit in the last few years. Therefore, we have now added some sentences discussing base-editing and HDR to change individual DNA bases in the section introducing CRISPR techniques.

4. The authors do a good job at the beginning highlighting that there is an important difference between cell lines transplant and autochthonous tumor models. It might be nice for the authors to go through the manuscript and make sure that this is clear for most of the examples that they highlight. I think that this is an important distinction.

- Thank you. In light of this, we have checked all examples given throughout the paper and have described in each case whether the model used was based on transplant or was autochthonous, given the importance of this distinction.

5. Suggesting some caution around genome scale in vivo screen could we warranted. The math around maintain representation in these screens is not always kept in mind even by the authors of those papers. I think that the limitation on the number of genes that can be interrogated in most in vivo CRISPR screens in a negative relative to transposon screens.

- Many thanks. This is an excellent point, and we agree there can be substantial difficulty in maintaining genome-wide library coverage *in vivo*, and here transposon mutagenesis screens provide an advantage. We have added this point to the paper in the section comparing the two techniques.

6. The section on CRISPR approaches in drug responses to treatments is pretty minimal. I imagine that this is because that data using these approaches in vivo have been limited. There are 2 manuscripts on Biorxiv where CRIPSR/CAs9 screens have been used to create diverse lung tumor genotypes followed by drug treatments (<https://www.biorxiv.org/content/10.1101/2020.04.13.036921v1> and <https://www.biorxiv.org/content/10.1101/2020.01.28.923912v1>). I am not sure whether these are worth citing or not.

- Thank you. Although this is an emerging important tool for drug resistance studies, there are relatively few published papers on CRISPR *in vivo* screens in this area. We appreciate the second reference given here, which is particularly relevant and has now been cited along with a brief discussion of the paper.

7. I don’t entirely agree with the statement referencing refs 146, 147. Those drugs were highly selected etc.

- Many thanks. A comment on this has been added to the review, making this point more measured.

8. The logic to outline a dual sgRNA screen in Figure 2 is unclear. I sort of field like a single sgRNA screen in more “typical”

- Thank you. It is certainly true that dual sgRNA screens are less typical than the single sgRNA ones; we have modified Figure 2 to show a single sgRNA screen instead.

This is really a very thoughtful and interesting review.
